# Chronic lymphocytic leukemia management in India: balancing evidence, access, and affordability in clinical practice

**DOI:** 10.3389/fonc.2026.1853089

**Published:** 2026-07-15

**Authors:** Shubham Sahni, Lata Rani, Ritu Gupta, Ajay Gogia

**Affiliations:** 1 Department of Medical Oncology, Dr. B. R. A. Institute Rotary Cancer Hospital (IRCH), All India Institute of Medical Sciences (AIIMS), New Delhi, India; 2 Genomics Lab, Centralized Core Research Facility (CCRF), All India Institute of Medical Sciences, New Delhi, India; 3 Laboratory Oncology Unit, Dr. B. R. A. Institute Rotary Cancer Hospital (IRCH), All India Institute of Medical Sciences (AIIMS), New Delhi, India

**Keywords:** BTK inhibitors, chemoimmunotherapy, chronic lymphocytic leukemia, cost-effectiveness, India, minimal residual disease, venetoclax

## Abstract

Management of Chronic lymphocytic leukemia (CLL) has undergone a paradigm shift, moving from chemoimmunotherapy (CIT) to precision, biology-driven treatment with targeted agents. While these advances have rapidly reshaped practice in high-resource nations, their integration into routine care in India is limited by differences in epidemiology, healthcare infrastructure, drug access, and economic constraints. CLL remains relatively uncommon in India, with patients presenting at a younger age and historically at more advanced stages of disease. Nevertheless, there is a temporal shift towards earlier diagnosis with the wider use of hematological testing. Risk stratification has also evolved from clinical staging to molecular profiling incorporating TP53 mutation testing, IGHV mutation status, and cytogenetic abnormalities. However, access to molecular profiling remains limited across India, directly influencing therapeutic decision-making. Bruton tyrosine kinase inhibitors (BTKi) and BCL-2 inhibitors have demonstrated superior efficacy and tolerability compared to CIT across biological subgroups and are endorsed by international guidelines. In India, treatment selection continues to balance biological risk with access and affordability. The introduction of generic ibrutinib has been important, especially for TP53 mutated CLL, while uptake of venetoclax-based fixed-duration regimens has been limited by cost and monitoring requirements. CIT retains a limited role in selected young, IGHV-mutated patients with durable responses. This review examines contemporary CLL management through an Indian lens, emphasizing how global advances intersect with real-world constraints and influence local practice. Improving access to molecular diagnostics and targeted agents along with health-policy reforms is essential to narrow the outcome gap between Indian and Western patients with CLL.

## Introduction

Chronic lymphocytic leukemia (CLL) is the most common adult leukemia in Western countries, but in India it remains relatively uncommon, accounting for ~5% of leukemias in India. However, diagnoses of CLL in India have increased over the past decade due to better awareness and access to diagnostics (1–3). Indian institutional cohorts have reported younger age at presentation and a higher proportion of symptomatic or advanced stage disease compared with Western series; however, these observations may reflect referral bias, delayed diagnosis, healthcare-access barriers, and incomplete detection of asymptomatic early-stage disease, in addition to possible biological differences (2). Over the past decade, CLL management has undergone a paradigm shift from uniform chemoimmunotherapy to precision, biology-driven targeted therapy with novel agents such as Bruton tyrosine kinase inhibitors (BTKi) and BCL-2 inhibitors, markedly improving depth of remission and tolerability (4). These advances are increasingly reshaping Indian practice, despite persistent challenges of cost and access. A review specific to Indian practice is warranted as CLL care in India differs from Western practice not only in epidemiology and presentation, but also in diagnostic access, treatment affordability, public-private sector availability of targeted agents, insurance coverage, and feasibility of molecular testing, minimal residual disease (MRD) assessment, venetoclax ramp-up monitoring, and long-term toxicity surveillance. This review examines the evolving therapeutic landscape of CLL from an Indian perspective, highlighting how local constraints that are unique to low- and middle-income countries (LMICs) shape therapeutic paradigms in India.

## Epidemiology and presentation

Incidence of CLL varies strikingly by geography and ethnicity. It is essentially a disease of older adults, with an age-adjusted incidence ~4–5 per 100,000 in the West (4). In contrast, population-based analysis in India reported much lower incidence of approximately 0.14 per 100,000 (1, 5). Indian patients are consistently a decade younger at presentation compared with the West (2).

Owing to broader use of routine laboratory testing and health screenings to detect asymptomatic lymphocytosis, there is a temporal trend toward earlier diagnosis. In Western cohorts, over 70% of CLL patients are asymptomatic at diagnosis (Binet stage A/Rai 0) and are managed under observation without treatment (6). Indian cohorts traditionally had more advanced stages at presentation (7). However, in a recent real-world dataset from North India, 42.3% of patients were asymptomatic, identified via incidentally detected lymphocytosis on routine hemograms. Nevertheless, symptomatic presentations remained common: for example, in one cohort 28.6% had lymphadenopathy, 14.9% had infections and, 14.2% had B symptoms at diagnosis (1). **Table 1** summarizes the demographic and clinical characteristics of CLL patients reported in Indian studies (1, 2, 7–12).

**Table 1:** Demographic and clinical details of CLL patients enrolled in various Indian studies.

Study	N	Study design	Median age (Years)	Male (%)	No symptoms (%)	Lymphadeno-pathy (%)	Fatigue (%)	B (%)	Hepatomegaly (%)	Splenomegaly (%)	Hb (g/dL)	TLC (10^9^/L)	ALC (10^9^/L)	Platelet count (10^9^/L)	Rai staging
Gogia et al, 2012 (8)	285	Retrospective observational	59	73	22	43	30	20	40.3	50.9	11.5	50	40.9	150	0: 10%, I: 16.1%, II: 33.3%, III: 20%, IV: 21.1%
Agarwal et al, 2007 (7)	95	Retrospective observational	61	78.90	7.4	55	–	25	63	66	–	70.6	51.5	–	0: 16.8%, I: 8.4%, II: 45.2%, III: 11.5%, IV: 17.8%
Gogia et al, 2020 (9)	36 (Del 17p)	Prospective observational	57	83.3	–	–	–	–	–	–	11.5g	–	55.9	110	0: 0%, I: 13.8%, II: 27.78%, III: 22.22%, IV: 36.11%
Gogia et al, 2019 (10)	16 (De-novo del 17p)	Retrospective observational	53	73.3	–	–	–	–	–	–	11	60	52	130	0: None, I: 18.75%, II: 31.25%, III: 18.75%, IV: 31.25%
Gogia et al, 2014 (11)	222 (Untreated)	Retrospective observational	60	75	4.5	40	30	20	35	45	11.8	48	42	160	0: 6%, I: 19%, II: 39%, III: 15%, IV: 20%
Kaur et al, 2020 (12)	89	Retrospective observational	60	76.5	–	–	–	–	–	–	–	–	–	–	0: 16.8%, I: 15.7%, II: 31.4%, III: 14.6%, IV: 21.3%
Tejaswi et al, 2020 (1)	409	Prospective observational	61	70.6	42.3	28.6	–	14.2	–	–	–	–	–	–	0: 12%, I: 20%, II: 25.4%, III: 20.8%, IV: 21.8%
Jacob et al, 2025 (2)	63	Retrospective observational	60	54	11.1	71.6	38.1	36.5	84.1	58.7	11.1	71.3	60.6	80	0: 4.8%, I:11.1%, II:25.4%, III:17.5%, IV:41.3%,

ALC, Absolute lymphocyte counts; B, B-symptoms; Hb, Haemoglobin; No symptoms., No symptoms at presentation/Incidentally detected; TLC, Total Leukocyte count.

Although several Indian institutional cohorts suggest younger age at presentation, higher disease burden, and shorter time to treatment compared with Western cohorts, these observations should be interpreted cautiously. The available Indian data are largely derived from tertiary referral centers and institutional cohorts, and not population-level registries. As a result, data may be influenced by referral bias, delayed recognition of asymptomatic lymphocytosis, variable access molecular testing, and socioeconomic barriers to early specialist care. True biological differences cannot be excluded, particularly given the younger age at presentation and cytogenetic differences reported in some Indian cohorts. However, current evidence is insufficient to conclusively attribute these differences to inherently aggressive CLL biology. Prospective population-level Indian datasets incorporating standardized staging, FISH, TP53 and IGHV testing, treatment exposure, and longitudinal outcomes are needed to clarify whether observed differences reflect biology, healthcare access, referral patterns, or a combination of these factors.

## Diagnosis and initial work-up

The diagnosis of CLL is established by peripheral blood flow cytometry demonstrating a characteristic immunophenotype (13). The updated iwCLL criteria (4) require an absolute B-lymphocyte count ≥5 × 10⁹/L in peripheral blood for ≥3 months (or <5 × 10⁹/L if lymph node biopsy confirms small lymphocytic lymphoma). Typical immunophenotyping panels in India include markers to differentiate CLL from other lymphoproliferative disorders, especially mantle cell lymphoma. Given resource constraints, many centers perform a limited panel (CD5, CD19, CD20, CD23, CD10, CD3, κ/λ) which is sufficient to confirm CLL in most cases. In atypical or borderline cases, additional markers (e.g. FMC7, CD200) or molecular tests for *CCND1* translocation are used to exclude mantle cell or other entities.

Once diagnosed, baseline work-up as per guidelines should include a thorough clinical evaluation, blood counts and chemistries, direct antiglobulin test (Coombs test for autoimmune hemolysis), β2-microglobulin level, and infectious screenings (hepatitis B/C, HIV) prior to therapy initiation. Staging by Rai or Binet classification is done, though its prognostic relevance has been eclipsed by molecular markers. Fluorescence-*in-situ* hybridization (FISH) for del(17p), del(13q), trisomy 12 and del(11q), as well as sequencing for IGHV and TP53 mutations, is recommended at diagnosis. A bone marrow examination is usually not needed except to evaluate unexplained cytopenias. Cross-sectional computed tomography (CT) scans of the chest and/or abdomen may be obtained when bulky lymphadenopathy is suspected. Positron emission tomography/computed tomography (PET/CT) is valuable in CLL patients with progressive or atypical disease features to identify lesions suspicious for Richter transformation or second malignancy, and to guide optimal biopsy site selection (14).

## Prognostic and predictive biomarkers in CLL

Risk stratification in CLL has evolved from simple clinical staging to a sophisticated array of molecular and cytogenetic prognostic markers. The key prognostic biomarkers have similar significance worldwide, but their use is constrained in India by cost and limited laboratory infrastructure.

### Cytogenetic abnormalities (FISH)

Chromosomal aberrations detected by FISH are important predictors of outcome. Across major Indian series, 70% of patients harbor at least one cytogenetic abnormality (1). Del(13q) is the most common abnormality (~50% cases) and confers a favorable prognosis when isolated. Del(11q) occurs in ~10–20% and portends more aggressive disease with extensive lymphadenopathy and shorter survival. Trisomy 12 (~15–20%) is intermediate risk. The highest-risk lesion is del (17p), involving the TP53 tumor suppressor, found in ~5–10% of treatment-naïve CLL in the West and up to 30–40% of relapsed/refractory cases (15). Indian data suggest a ~12% prevalence of del(17p) among newly diagnosed patients, substantially higher than Western estimates, likely due to referral bias toward advanced disease (9). However, FISH testing is not universally performed in India due to financial and logistic barriers (16). In a large North Indian real-world cohort, FISH testing was available in 53.3% of newly diagnosed patients at baseline, reflecting incomplete uptake even at tertiary centers (1). Resource constraints (and lack of impact if novel agents are unaffordable) mean some patients are treated empirically without full risk stratification. Integration of FISH into standard diagnostics in India is improving with increasing physician awareness and broader availability of molecular diagnostics. Nevertheless, most experts in India would at minimum obtain FISH for del(17p), as it is critical to avoid chemoimmunotherapy in these patients.

### TP53 mutation

TP53 mutation is one of the strongest predictors of poor outcome. This aligns with global biology but carries distinct clinical implications in India: if TP53 testing is not performed, many patients risk receiving futile chemoimmunotherapy. Indeed, the emergence of generic ibrutinib has made TP53 testing more actionable, since an increasing number of Indian patients can be offered a BTK inhibitor if rendered high-risk by molecular profiling.

### IGHV mutation status

IGHV mutation status remains a cornerstone prognostic and predictive biomarker (17, 18). Mutational analysis is performed via Sanger sequencing of DNA or complementary DNA (cDNA) and compared with germline sequences in immunoglobulin databases. Current evidence suggests that IGHV-mutated CLL has potential for long-term remissions with chemoimmunotherapy, whereas IGHV-unmutated CLL derives increased benefit from novel therapies (19). Testing rates in India are low, primarily due to cost and limited sequencing facilities.

### CLL-IPI

The CLL International Prognostic Index (CLL-IPI) (20) is a validated tool for risk stratification in CLL. It integrates five prognostic variables: TP53 status (del(17p)/mutation), IGHV mutation status, serum β2-microglobulin, clinical stage (Rai/Binet), and age. CLL-IPI has demonstrated consistent ability to predict overall survival (OS). In a prospective cohort of 198 treatment-naïve CLL patients from India, CLL-IPI was compared against other prognostic models (21). CLL-IPI demonstrated the best prognostic discrimination, outperforming other models. This study validated CLL-IPI as the most robust prognostic tool for Indian CLL patients, supporting its routine incorporation into risk stratification and management planning.

## Indian context and guideline implementation

Beyond affordability alone, the implementation of modern CLL care in India is shaped by where a patient enters the healthcare system. Patients evaluated at academic or private tertiary centers are more likely to have access to expert hematopathology review, molecular risk stratification, structured toxicity monitoring, venetoclax ramp-up protocols, and newer targeted agents. In contrast, patients initially seen in smaller hospitals or non-metropolitan settings may undergo delayed referral, incomplete baseline risk assessment, or treatment decisions based primarily on clinical stage, symptoms, and immediate drug availability. Public tertiary centers often provide high-volume specialist expertise and relatively affordable chemotherapy, rituximab/biosimilars, transfusion support, and infection management, but access to continuous targeted therapy may be limited by formulary restrictions, reimbursement pathways, and patient-assistance mechanisms. Private-sector care may offer broader access to BTK inhibitors, venetoclax-based regimens, second-generation BTK inhibitors, and outsourced molecular testing, but at the cost of substantial out-of-pocket expenditure. Therefore, the Indian treatment decision is frequently determined not only by disease biology, but also by referral pathway, geography, payer status, and monitoring feasibility.

Indian guidelines include observation for patients without treatment indications, FCR for selected young and fit patients without 17p deletion, BR or ibrutinib for elderly or comorbid patients, and ibrutinib-based therapy for patients with 17p deletion or p53-mutated disease. This framework remains useful because it reflects an access-sensitive approach suited to routine Indian practice. However, more recent guidelines predates several contemporary developments, including wider use of venetoclax-obinutuzumab, second-generation BTK inhibitors, MRD-guided fixed-duration therapy, and non-covalent BTK inhibitors. In daily practice, Indian clinicians therefore often integrate expert guidance with iwCLL treatment-initiation criteria and contemporary international evidence, while adapting final decisions to biologic risk, drug availability, affordability, and the feasibility of safe monitoring.

## Timing of therapy initiation in CLL

A crucial aspect of CLL management is recognizing that not all patients need immediate therapy. The iwCLL (International Workshop on CLL) guidelines enumerate specific indications for treatment, often called the “active disease” criteria. Patients who do not meet these criteria should be managed with observation (“watch and wait”), regardless of risk markers (22). Given the heterogeneous natural history of CLL, treatment of early-stage disease (as defined by iwCLL) provides no advantage (22). This paradigm has been confirmed even with ibrutinib (23, 24). Adherence to “no treatment without indication” is generally practiced in India as well. An important consideration is to perform prognostic testing (FISH for del(17p), TP53 mutation, and IGHV) before starting first-line therapy, because choice of regimen hinges on these results.

## Treatment landscape of CLL

The treatment landscape of CLL has transformed significantly over the last two decades, moving away from chemoimmunotherapy (CIT), which had acceptable efficacy in a small subset (notably IGHV-mutated CLL) but limited efficacy in high-risk disease (TP53 aberrant or IGHV-unmutated). The advent of oral targeted agents, notably Bruton tyrosine kinase (BTK) inhibitors (ibrutinib, acalabrutinib, zanubrutinib) and BCL-2 inhibitor venetoclax, has revolutionized CLL therapy. These agents have demonstrated superior survival estimates compared to CIT, leading to their rapid adoption in Western guidelines as first-line therapy for most patients. Below we outline the major treatment modalities and their evidence, then contrast how these therapies are applied in India owing to local constraints.

### Chemoimmunotherapy

The combination of chemotherapy with anti-CD20 monoclonal antibodies was the cornerstone of CLL treatment in the 2000s. In the pivotal CLL8 trial, fludarabine, cyclophosphamide and rituximab (FCR) demonstrated impressive responses and improved survival compared to FC alone (25). The benefit of FCR was consistent across most subgroups except del (17p). The most durable outcomes were seen in IGHV-mutated CLL. FCR produced durable disease control, with 5-year progression-free survival (PFS) of 66.6% vs 36.2% with FC, suggesting a functional cure in a subset. However, FCR is highly myelotoxic and poorly tolerated in older or comorbid patients, with a high risk of infections and therapy-related myeloid neoplasms. Bendamustine-rituximab (BR) has emerged as a less toxic alternative for patients unsuitable for FCR. In CLL10 trial, FCR demonstrated higher CR and PFS than BR in fit patients, but with similar overall survival (OS) and greater toxicity (19). For frail patients, the historical standard was chlorambucil, which was later improved by adding an anti-CD20 antibody (CLL11) (26).

The efficacy of CIT is biology-dependent. Durable remissions are largely confined to IGHV-mutated, TP53-intact CLL. Outcomes are inferior in IGHV-unmutated disease and del(17p)/TP53-mutated CLL, in whom chemotherapy is biologically ineffective (19, 25, 27). Patients with del(17p) or TP53 mutations derive minimal benefit from CIT because DNA-damage-dependent cytotoxicity is ineffective when p53-mediated apoptosis is defective (15, 28). Consequently, major CIT trials either excluded these patients or showed uniformly poor outcomes when they were included (19, 25, 26). As a result, CIT has largely been displaced in the West, with targeted agents now favored across all ages and IGHV categories when accessible. This shift reflects the consistently superior depth of response, tolerability, and long-term disease control achieved with targeted therapies.

In India, CIT has remained relevant for a longer period due to cost constraints. **Table 2** summarizes the therapeutic patterns and outcomes of frontline CLL from Indian studies (1, 2, 7–10). Early Indian series used chlorambucil- or fludarabine-based therapies, with limited efficacy. In a North Indian series, chlorambucil achieved an objective response rate (ORR) of 69% with only 3% complete response (CR), while fludarabine-based therapy produced higher responses (ORR 89%, CR 44%) but substantial toxicity (febrile neutropenia, prolonged myelosuppression and opportunistic infections) (8). In the recent survey, most Indian hematologists reported that FCR is rarely used as first-line CIT in India because of the high incidence of grade 3–4 cytopenias, prolonged neutropenia, infections, and the requirement for intensive supportive care (3). These factors limit its use outside major centers. More recent real-world Indian cohorts treated predominantly with BR demonstrate deeper remissions (CR/CRu 77.2%) and a high ORR (91.2%), establishing BR as the preferred CIT in India (1, 8, 11). For elderly or frail patients who cannot afford novel agents, chlorambucil-prednisolone ± rituximab remains a palliative option.

**Table 2:** Studies detailing treatment patterns and outcomes for frontline CLL in India.

Study	N	Study design	Proportion treated	First-line therapy	ORR%	CR/CRu	Median follow-up	Median OS	Median EFS
Gogia et al, 2012 (8)	285	Retrospective observational	49.47%	Oral chlorambucil (68%), FCR (19%), CVP or CHOP (14%)	Chlorambucil: 68.7%, FCR: 88.88%, CVP/CHOP: 71%	Chlorambucil: 3.1%, FCR: 44.44%, CVP/CHOP: 7.0%	2.9 years	Overall: 5.1 years, Rai 0/1: NR, Rai 2: 8.2, Rai ¾: 3.2	Overall: 4.6, Rai 0/1: 8.2, Rai 2: 6.4, Rai ¾: 2.9
Agarwal et al, 2007 (7)	95	Retrospective observational	55.70%	Chlorambucil: 53/95, Fludarabine: 18/95 (Outcomes reported for Chlorambucil only)	11.3%	0%	–	4 years	–
Gogia et al, 2020 (9)	36 (Del 17p)	Prospective observational	De-novo: 61.1%, R/R: 83.3%	BR: 30.7%, CP: 15.3%, Ibrutinib: 30.7%, FCR: 33.33%, RCHOP/RCVP: 19.2%	50%	23%	29 months	5-year OS: 40%	14 months
Gogia et al, 2019 (10)	16 (De-novo del 17p)	Retrospective observational	62.50%	BR: 40%, FCR: 40%, CP: 10%, Ibrutinib: 40%	–	–	–	–	–
Gogia et al, 2014 (11)	222 (None on treatment)	Retrospective observational	45%	Chlorambucil based: 141, FCR: 37, CVP/CHOP: 14	Chlorambucil based: 66%, FCR: 88%	Chlorambucil based: 3%, FCR: 40%	3.5 years	5 years	–
Tejaswi et al, 2020 (1)	409	Prospective observational	58.70%	CP: 52.2%, BR: 31.6%, FCR: 3.3%, R-Clb: 3.8%, CVP/CHOP: 4.4%, Ibrutinib (N = 4)	CP: 74.5%, BR: 91.2%	CRu rates CP: 3.2%, BR: 77.2%	32 months	Not reached	Median TTNT BR: NR, CP: 33 months
Jacob et al, 2025 (2)	63	Retrospective observational	88.9%	BR 42.8%; Chorlambucil+Wysolone 28.5%; Ibrutinib 17.4%; Observation 11.1%	–	28.5%	17 months	Not reached	17.6 months

BR, Bendamustine – rituximab; CHOP, Cyclophosphamide – Doxorubicin – vincristine – prednisolone; CVP, Cyclophosphamide – vincristine – prednisolone; CR, Complete response; CRu, Unconfirmed complete response; EFS, Event free survival; FCR, Fludarabine – cyclophosphamide – rituximab; ORR, Overall response rate; OS, Overall survival; TTNT, Time to next treatment.

### BTK inhibitors

The introduction of ibrutinib, an irreversible oral inhibitor of Bruton’s tyrosine kinase, marked the first major breakthrough in CLL therapy since rituximab. Ibrutinib blocks B-cell receptor (BCR) signaling, leading to CLL cell apoptosis and egress from lymphoid tissues. In the pivotal RESONATE trial, ibrutinib significantly improved PFS and OS compared to ofatumumab in relapsed CLL, and subsequent frontline trials confirmed its superiority over CIT (29). E1912 trial showed that ibrutinib–rituximab (IR) improved PFS and OS versus FCR in young, fit patients, while Alliance A041202 and iLLUMINATE showed improved PFS and tolerability over BR or chlorambucil-based regimens in older patients (30–32). Notably, adding rituximab to ibrutinib did not improve outcomes over ibrutinib alone. Collectively, these studies establish continuous BTK inhibition as the preferred frontline strategy across most clinical and biological subgroups, including high-risk CLL, positioning BTK inhibition as a cornerstone of modern CLL therapy.

### Next-generation BTK inhibitors

Ibrutinib’s success is tempered by adverse effects, notably atrial fibrillation, hypertension, bleeding tendency, rash, arthralgias, and diarrhea (33). Many side effects are related to off-target kinase inhibition. Acalabrutinib and zanubrutinib were developed as more selective second-generation BTK inhibitors with reduced toxicity (34). In randomized trials, acalabrutinib-based therapy significantly improved PFS over chlorambucil in frontline CLL (ELEVATE-TN) and showed non-inferior efficacy with a superior cardiovascular safety profile compared to ibrutinib in high-risk relapsed CLL (ELEVATE-RR) (35–37). Zanubrutinib showed superior PFS and fewer toxicities compared to ibrutinib in relapsed CLL (ALPINE) and improved PFS over BR in frontline CLL (SEQUOIA), collectively establishing second-generation BTK inhibitors as better tolerated alternatives to ibrutinib (38, 39).

### BTK inhibitor use in India

Ibrutinib has become an important novel agent in Indian CLL practice. A key step was the introduction of generic ibrutinib in India around 2019, which drastically reduced the cost from about ₹300,000/month (US ~$3,500) for the innovator molecule to ₹15,000/month (US ~$180) for generics. This has enabled many more patients to access therapy. Multiple centers across India now report clear patient preference for oral BTK inhibitors over CIT due to better tolerability, fewer hospital visits and improved quality of life (3). Challenges remain, including erratic availability of generic ibrutinib in India. Real-world Indian data highlight financial toxicity as the major barrier to ibrutinib use. Some patients have resorted to sustained reduced doses with comparable early outcomes to standard dosing, with fewer adverse events, though numbers are small (40). Indefinite ibrutinib therapy is a significant financial burden for patients and families. On the positive side, real-world tolerance of ibrutinib appears acceptable (41). Access to acalabrutinib and zanubrutinib remains limited due to high cost. As a consequence, ibrutinib remains the workhorse BTK inhibitor in India.

### Venetoclax and fixed-duration therapy

Venetoclax is a potent oral BCL-2 inhibitor that induces apoptosis in CLL cells, including chemotherapy-resistant clones. In relapsed CLL, the MURANO trial demonstrated that 2-year venetoclax-rituximab significantly improved PFS over BR and achieved high rates of undetectable MRD (uMRD) (42). In the frontline setting, the CLL14 trial showed improved PFS and MRD clearance with fixed-duration venetoclax-obinutuzumab across molecular subgroups, although absolute outcomes in the TP53-mutated subgroup remained inferior (43). The CLL13 trial further established venetoclax-obinutuzumab as an effective fixed-duration regimen for fit, TP53-unmutated CLL, providing deeper and more durable remissions, compared to CIT (44). The greatest benefit was seen in IGHV-unmutated CLL, while IGHV-mutated CLL outcomes were similar to those achieved with FCR.

More recently, combination targeted therapy has been used in trials like CAPTIVATE and GLOW, demonstrating high rates of MRD clearance and durable PFS with ibrutinib-venetoclax based regimen compared to FCR (45, 46). In the CAPTIVATE trial, this regimen achieved a CR rate of 56%, uMRD rates of 77% in blood and 60% in marrow, and 24-month PFS and OS of 95% and 98%, respectively, with consistently high efficacy even in high-risk subsets (del(17p)/TP53-mutated and IGHV-unmutated). The phase 3 GLOW trial found that ibrutinib-venetoclax-obinutuzumab for 12 cycles was superior in PFS to chlorambucil-obinutuzumab in older patients, establishing the proof-of-concept for chemotherapy-free combination therapy. Together, these studies underscore that fixed-duration, all-oral therapy with ibrutinib-venetoclax provides a highly effective, time-limited approach applicable across diverse CLL populations.

### Cost versus cure: assessing the utility of available therapies in the Indian context

Cost remains an important barrier to optimal CLL care in India. A landmark cost-effectiveness analysis by Nehra et al. evaluated chlorambucil-prednisolone (CP), bendamustine-rituximab (BR), and ibrutinib, using a Markov model based on data from six major Indian centers (5). At current prices, CP followed by BR was the most cost-effective strategy, whereas regimens incorporating ibrutinib were not cost-effective at the conventional Indian threshold (1× per capita GDP per QALY), unless drug prices declined by >80%. These findings explain why CIT, though obsolete globally, is likely to persist in Indian practice until there is broader insurance coverage or major price reductions for novel agents. Inclusion of targeted agents in public-health reimbursement schemes would substantially reduce out-of-pocket expenditure and enable equitable access. In practical terms, this means that while ibrutinib has become common in private practice and in resource-rich centers in India, its incorporation into publicly funded institutions is still limited. Access to second-generation BTK inhibitors remains limited in India.

Despite improved efficacy with targeted agents, a sizeable minority may still benefit from frontline CIT given its accessibility, affordability, and finite duration. Apart from pragmatism, chemoimmunotherapy retains select advantages. Firstly, these agents have the longest follow-up data demonstrating efficacy and safety (27). Secondly, up to 70% of patients with mutated IGHV show long-lasting remissions with FCR (median follow-up: 12.8 years). This makes FCR the only evidence-based frontline option offering potential “cure”. Third, most salvage options have been validated in a CIT-pretreated population (29, 42). Lastly, improvement over frontline CIT in IGHV-mutated patients is yet to be demonstrated with novel agents. Thus, CIT may still be the therapy of choice for a fit, young patient with mutated IGHV. However, the following considerations argue against CIT in this setting. Firstly, the introduction of generic formulations of targeted agents may narrow the financial toxicity gap. Secondly, long-term survivors with CLL treated with CIT are at risk of developing therapy-related myeloid neoplasms (47). Third, CIT recipients show alarmingly high rates of subclonal and clonal high-risk mutations (especially in IGHV- unmutated CLL) (48, 49). Although ibrutinib is given indefinitely, adverse effects tend to decline over time, and the persistent effects can be managed medically. Acalabrutinib may further improve tolerability (37). In prospective randomized trials, ibrutinib has shown sustained improvements in quality of life along with more frequent independence from transfusions (50).

**Figure 1:**
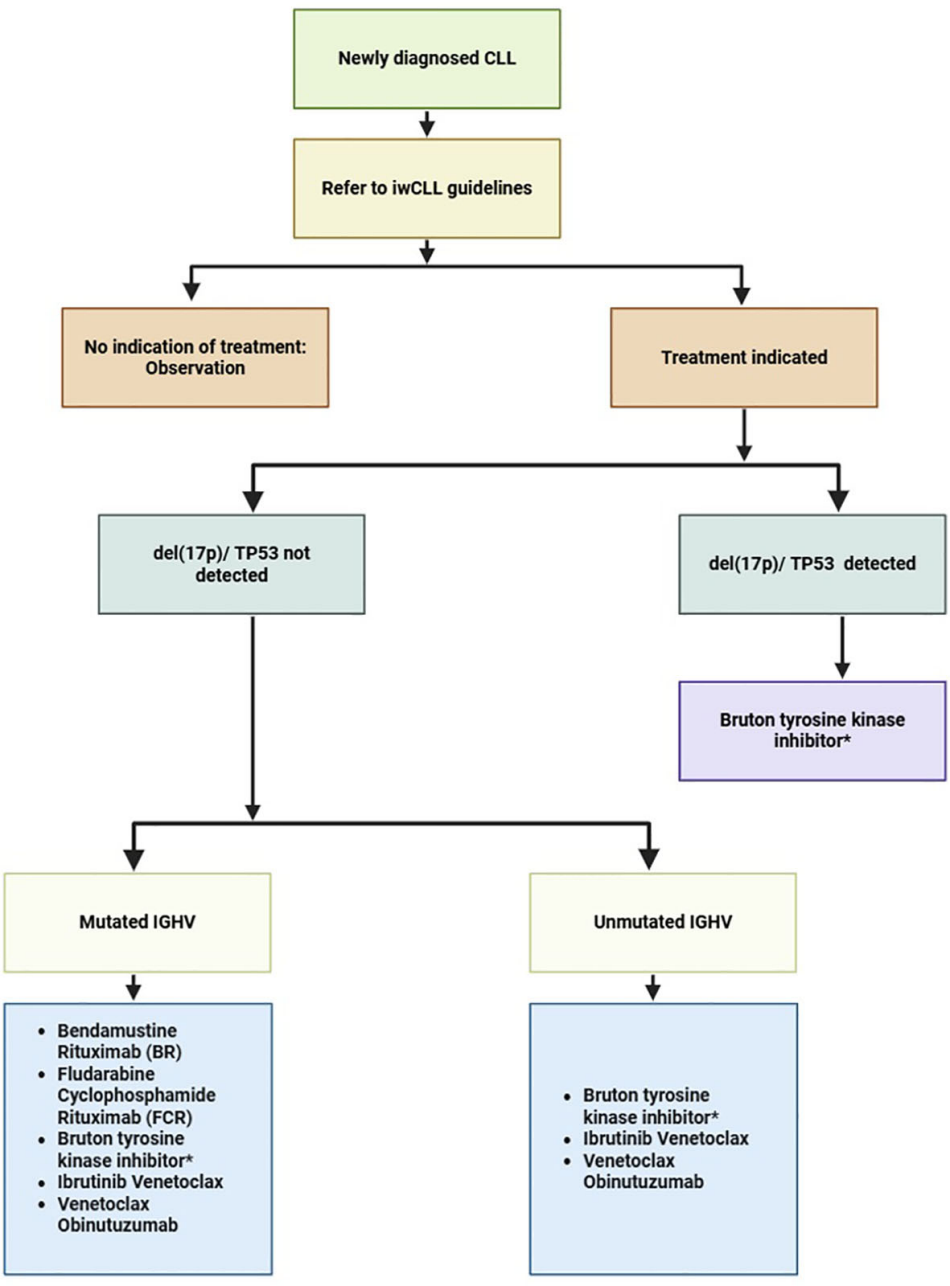
Practical frontline treatment pathway for CLL applicable to Indian practice. Frontline treatment algorithm for chronic lymphocytic leukemia in India. Treatment should be initiated only when iwCLL criteria are met. Choice of therapy should incorporate TP53 status, IGHV mutation status, patient fitness, drug availability, affordability, and feasibility of monitoring. This author-developed, evidence-informed pathway represents the authors’ expert opinion and are not reproductions of an official national guideline. Indian National Cancer Grid guidance for CLL exists but predates several contemporary therapies; the proposed pathways in the figure are based on iwCLL criteria, current international evidence, Indian real-world data, and practical considerations of access, affordability, and feasibility. ^iwCLL, International Workshop on Chronic Lymphocytic Leukemia guidelines; *Obinutuzumab may be added to BTKi when affordable; BTKi, Bruton Tyrosine Kinase inhibitor; del17p, deletion 17p.

**Figure 1** presents a practical frontline treatment algorithm for CLL in India. The algorithm begins with iwCLL treatment-initiation criteria and then incorporates TP53 status, IGHV mutation status, and practical treatment availability It is intended as a pragmatic framework rather than a rigid guideline, recognizing that treatment selection in India is influenced not only by disease biology but also by drug access, affordability, patient preference, and feasibility of long-term monitoring.

### Translating global evidence into Indian practice

Global CLL trials have established targeted agents as preferred therapy for most biological subgroups; however, their direct translation to Indian practice requires caution (3). Patients enrolled in pivotal trials are typically treated in systems with structured follow-up, reliable molecular testing, standardized tumor lysis syndrome (TLS) monitoring, drug reimbursement, and prompt management of treatment-emergent toxicities (19, 50). In India, clinical decisions are often shaped by delayed presentation, variable access to TP53 and IGHV testing, differences in drug availability, limited insurance coverage, and the need to minimize out-of-pocket expenditure (1, 5). Continuous BTK inhibition is attractive because of oral administration, avoidance of chemotherapy-related myelosuppression, and the availability of generic ibrutinib (51). However, indefinite therapy creates cumulative financial toxicity and requires long-term monitoring for hypertension, atrial fibrillation, bleeding, infections, and drug interactions (52). Conversely, venetoclax-based fixed-duration therapy offers the conceptual advantage of time-limited treatment and deep MRD-negative remissions, but it requires structured TLS risk assessment, dose ramp-up, frequent laboratory monitoring, and hospitalization for selected high-risk patients (53). Thus, the optimal Indian approach is not a simple replication of Western guidelines but a risk-adapted, access-sensitive strategy that balances disease biology, patient fitness, treatment duration, monitoring feasibility, and affordability.

These differences also influence real-world treatment adoption and outcomes. In routine Indian practice, treatment selection may be determined by the regimen that can be safely initiated, monitored, and financially sustained rather than by the globally preferred option (54). Interruption or discontinuation of continuous therapy due to cost, delayed recognition of adverse events, inconsistent follow-up, and limited access to specialized toxicity management may reduce the effectiveness observed in clinical trials (55). Therefore, applying global evidence in India requires a pragmatic framework that integrates efficacy with feasibility, continuity of access, toxicity surveillance, and patient-level financial sustainability.

### Minimal residual disease driven treatment: toward precision medicine

One of the most important advances with venetoclax-based therapy is the frequent achievement of uMRD. MRD negativity, assessed by multi-color flow cytometry or PCR/NGS to a sensitivity of 10^-4^ to 10^-5^, is strongly associated with prolonged PFS and OS in CLL and is now an established surrogate endpoint in fixed-duration regimens. The FLAIR trial provided the strongest proof-of-concept for MRD-adapted therapy in CLL. In this phase III study, MRD-adapted, fixed-duration combinations of ibrutinib-venetoclax improved 3-year PFS (97.2% vs 76.8%; HR 0.13) and OS (HR 0.31) compared to FCR in fit, treatment-naïve patients (56). Treatment decisions in the ibrutinib-venetoclax arm were strictly MRD-driven: peripheral blood MRD was measured at regular intervals, and patients who achieved confirmed uMRD at predefined timepoints discontinued all therapy after ~2 years, whereas those with detectable MRD continued ibrutinib (or both agents) as per protocol. Nearly half of patients met criteria for treatment cessation following sustained uMRD. The benefit was striking in IGHV-unmutated and TP53-aberrant subgroups, while IGHV-mutated patients achieved PFS comparable to FCR with substantially less myelosuppression and avoidance of therapy-related myeloid neoplasms. Collectively, FLAIR demonstrates that time-limited, all-oral therapy with ibrutinib-venetoclax delivers deep, durable, and broadly applicable remissions, redefining the frontline standard of care for fit, treatment-naïve CLL.

In India, MRD assessment is currently limited to clinical trials or specialized centers due to cost and technical requirements. Presently, only a minority of Indian CLL patients receive venetoclax because of cost and need for close TLS monitoring during initiation. In Western trials, TLS risk was mitigated by stepwise dose escalation over 5 weeks. Ensuring comparable monitoring with frequent laboratory tests and inpatient observation for high-risk cases in India’s public healthcare system is challenging. As a result, formal published Indian data on venetoclax are still awaited. Nevertheless, as venetoclax becomes more accessible through patient assistance programs or generics, it could transform CLL therapy in India by enabling time-limited therapies with deep remissions. The concept of MRD-driven therapy aligns well with the goal of minimizing both cost and cumulative toxicity.

## Special management considerations

### Management strategy for Del (17p) and/or TP53 mutated CLL

Patients with del (17p)/TP53 mutated CLL derive little benefit from CIT and should ideally receive targeted therapies. In the CLL8 trial, median PFS with FCR varied significantly by cytogenetics (31.4 months in del(11q), 50.4 months in normal cytogenetics, and 11.3 months in del(17p), underscoring the limited utility of CIT in this subgroup (29). Current guidelines recommend a targeted agent as first-line therapy (either continuous BTKi or venetoclax-obinutuzumab). In India, generic availability and lower cost make continuous ibrutinib the most practical option, supported by real-world data (9, 41). Venetoclax-based regimens are preferred when accessible, but financial and hospitalization requirements limit their widespread use.

### Management strategy for relapsed CLL

In relapsed CLL, sequencing of targeted agents mainly depends on prior therapy and drug availability. Treatment sequencing in relapsed/refractory CLL increasingly depends on both prior treatment exposure and the mechanism of failure. After frontline chemoimmunotherapy, a BTK inhibitor or venetoclax-based regimen is preferred when accessible. After venetoclax-based fixed-duration therapy, a covalent BTK inhibitor is generally favored if not previously used. Venetoclax rechallenge may be considered in selected patients who relapse after a durable treatment-free interval, but is less attractive in patients with refractory progression on venetoclax. After progression on a covalent BTK inhibitor, venetoclax-based therapy remains a standard option where feasible. In contrast, intolerance to one covalent BTK inhibitor without disease progression may allow switching to a more selective covalent BTK inhibitor if available.

Resistance biology is increasingly relevant to treatment sequencing. Progression on covalent BTK inhibitors is commonly associated with acquired BTK kinase-domain mutations, particularly BTK C481S, which impairs irreversible drug binding, or downstream PLCG2 alterations that reactivate B-cell receptor signaling. Venetoclax resistance may involve acquired BCL2 mutations, including G101V, as well as broader adaptive mechanisms such as upregulation of alternative anti-apoptotic proteins and clonal evolution. Patients exposed to both BTK and BCL2 inhibitors represent a particularly difficult group with limited standard options. Non-covalent BTK inhibitors such as pirtobrutinib can inhibit BTK independent of the C481 binding site and have shown activity after covalent BTK inhibitor failure. In India, however, these sequencing principles must be adapted to drug access, affordability, patient fitness, monitoring feasibility, and goals of care, because many patients may realistically receive only one novel agent during the disease course.

**Figure 2:**
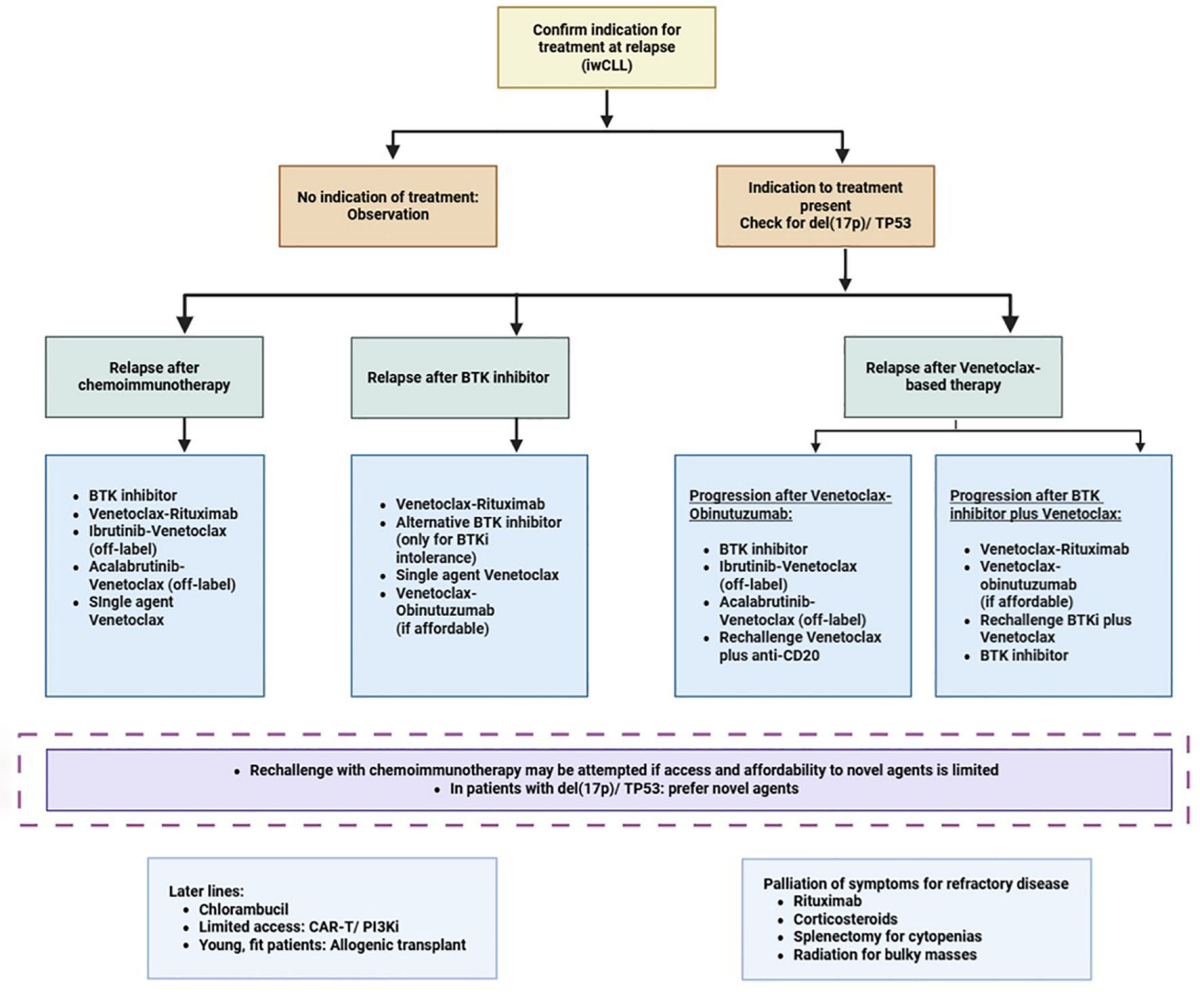
Pragmatic treatment algorithm for relapsed chronic lymphocytic leukemia in low- and middle-income countries. Pragmatic treatment algorithm for relapsed/refractory chronic lymphocytic leukemia in India and other resource-constrained settings. Treatment selection should consider iwCLL indication, repeat TP53 assessment, prior therapy exposure, mechanism of relapse or intolerance, access to BTK inhibitor- and venetoclax-based therapy, and goals of care. This author-developed, evidence-informed pathway represents the authors’ expert opinion and are not reproductions of an official national guideline. Indian National Cancer Grid guidance for CLL exists but predates several contemporary therapies; the proposed pathways in the figure are based on iwCLL criteria, current international evidence, Indian real-world data, and practical considerations of access, affordability, and feasibility. ^iwCLL, International Workshop on Chronic Lymphocytic Leukemia guidelines; *Obinutuzumab may be added to BTKi when affordable; BTKi, Bruton Tyrosine Kinase inhibitor; del17p, deletion 17p; CAR-T, Chimeric antigen receptor T-cell therapy; PI3Ki, Phosphoinositide 3-kinase inhibitors.

**Figure 2** provides a pragmatic relapsed/refractory CLL treatment algorithm tailored to LMIC practice. It emphasizes confirmation of iwCLL treatment indication at relapse, reassessment of del(17p)/TP53 status, consideration of prior therapy exposure, and sequencing between BTK inhibitor- and venetoclax-based strategies. In the Indian context, the algorithm also acknowledges that access to multiple sequential novel agents may be limited; therefore, treatment decisions must account for affordability, prior duration of response, toxicity, monitoring infrastructure, and goals of care.

### Chimeric antigen receptor T-cell therapy and other emerging therapies

The therapeutic landscape of CLL is continuing to evolve beyond covalent BTK inhibitors and venetoclax. Non-covalent BTK inhibitors, particularly pirtobrutinib, represent an important advance because they inhibit BTK independent of the C481 binding site and may retain activity after progression on covalent BTK inhibitors such as ibrutinib, acalabrutinib, or zanubrutinib (57). This class is especially relevant for patients progressing after covalent BTK inhibitor exposure, although access in India is currently expected to remain limited.

Cellular therapy has also entered the CLL treatment landscape. Lisocabtagene maraleucel, an anti-CD19 CAR-T-cell therapy, has demonstrated activity in heavily pretreated relapsed/refractory CLL/SLL after prior BTK inhibitor and BCL2 inhibitor exposure (58). However, CAR-T therapy in CLL is more challenging than in some aggressive B-cell lymphomas because patients are often older, heavily pretreated, immunosuppressed, and at risk for infections, prolonged cytopenias, cytokine-release syndrome, and immune-effector-cell-associated neurotoxicity syndrome. Therefore, cellular therapy is best viewed as an option for selected high-risk, fit, multiply relapsed patients treated at specialized centers rather than as a broadly applicable therapy in routine Indian CLL practice at present.

Bispecific antibodies are another emerging strategy. CD20×CD3 bispecific antibodies have transformed the management of several relapsed B-cell lymphomas and are being explored in CLL, particularly for heavily pretreated disease and patients previously exposed to both BTK and BCL2 inhibitors (59). Their use in CLL remains investigational and may be affected by disease-related T-cell dysfunction, infection risk, cytokine-release syndrome, need for step-up dosing, and requirement for close monitoring.

Fixed-duration combination regimens are likely to be more immediately relevant to future CLL practice than cellular therapy for most Indian patients. BTK inhibitor plus venetoclax-based combinations can produce deep remissions and high rates of undetectable MRD, with the potential advantage of limiting indefinite treatment exposure. However, these regimens still require access to both drugs, TLS monitoring, molecular risk stratification, and longitudinal MRD assessment if MRD-adapted strategies are used.

For India and other LMICs, the future relevance of cellular therapy will depend on cost reduction, local manufacturing, referral networks, toxicity-management infrastructure, and clinical-trial availability. Indigenous CAR-T platforms in India represent an important step toward improving access to cellular therapy for B-cell malignancies, although their routine application to CLL remains a future possibility rather than current standard practice. In the near term, the most practical Indian approach will likely involve wider access to affordable BTK inhibitors, gradual incorporation of venetoclax-based fixed-duration regimens where monitoring is feasible, and referral of selected high-risk or double-exposed patients to clinical trials, non-covalent BTK inhibitors, or cellular therapy programs where available.

### Follow-up and supportive care

Supportive care is central to CLL management and is particularly important in India, where infectious morbidity, delayed presentation, and variable access to monitoring may influence outcomes as much as antineoplastic therapy. Baseline screening before treatment should include hepatitis B surface antigen (HBsAg), antibody against hepatitis B core antigen (anti-HBc), hepatitis C serology (anti-HCV), human immunodeficiency virus (HIV), and assessment for tuberculosis risk, especially before anti-CD20 antibodies, prolonged corticosteroids, or targeted therapies associated with immune dysfunction. Patients receiving anti-CD20 antibodies require hepatitis B prophylaxis or close viral monitoring according to serologic status. Pneumocystis jiroveci pneumonia (PJP) and herpes zoster prophylaxis should be considered in patients receiving purine analogues, prolonged steroids, PI3K inhibitors, or heavily immunosuppressive regimens.

Vaccination should ideally be optimized before therapy and should include influenza, pneumococcal, COVID-19, and other age-appropriate non-live vaccines. Live vaccines should generally be avoided in immunocompromised patients. Recurrent severe bacterial infections in the setting of hypogammaglobulinemia may warrant IVIG replacement when feasible. Patients receiving BTK inhibitors require monitoring for hypertension, atrial fibrillation, bleeding, infections, and drug interactions, while venetoclax requires structured TLS risk assessment, hydration, dose ramp-up, and laboratory surveillance. Standardizing supportive-care pathways may substantially improve real-world outcomes in Indian CLL practice.

## Discussion

The management of CLL is undergoing a remarkable transformation, and India is steadily entering the era of precision, biology-driven therapy. While global treatment paradigms increasingly rely on TP53/IGHV-directed decision-making and novel targeted agents, India continues to face a gap between therapeutic possibilities and practical feasibility. Yet this gap is narrowing: the advent of affordable generic ibrutinib has already reshaped national practice, and emerging Indian real-world data demonstrate efficacy comparable to Western cohorts (1, 41). Innovations such as surrogate prognostic markers, expanding testing, and evolving health-policy frameworks are further enabling access (60).

Sustained progress will require coordinated efforts across oncologists, industry, government, and patient groups to ensure inclusion of targeted agents in public insurance schemes, strengthen local generic/biosimilar manufacturing, and expand India’s clinical-trial ecosystem to bring CAR-T, novel agents, and fixed-duration regimens within reach. Educational initiatives are also improving awareness of modern prognostic work-ups and MRD-based response assessment.

Overall, CLL in India is evolving from a historically under-recognized illness to a disease where personalized care is increasingly achievable. With ongoing research, cost reductions, and system-level strengthening, the long-term goal is to ensure that an Indian patient diagnosed with CLL has outcomes approaching those of their Western counterparts. The trajectory is positive, the therapeutic paradigms are converging, and continued progress promises to make CLL a chronic, manageable condition for patients across India.
